# 2-Methyl-1,2,3,4-tetra­hydro­isoquinolin-6-yl *N*-phenyl­carbamate

**DOI:** 10.1107/S1600536809013415

**Published:** 2009-04-18

**Authors:** Qi-Hong Zhang, Qiong Xie, Jing-Mei Wang, Zhui-Bai Qiu

**Affiliations:** a Department of Medicinal Chemistry, School of Pharmacy, Fudan University, 138 Yixueyuan Road, Shanghai 200032, People’s Republic of China; b Center of Analysis & Measurement, Fudan University, 220 Handan Road, Shanghai 200433, People’s Republic of China

## Abstract

In the mol­ecule of the title compound, C_17_H_18_N_2_O_2_, the piperidine ring adopts a half-chair form. The two benzene rings are individually planar and make a dihedral angle of 53.90°. The crystal structure is stabilized by inter­molecular N—H⋯N hydrogen bonds and π–π stacking inter­actions (centroid–centroid distance = 3.962 Å).

## Related literature

For a related structure, see: (Li *et al.*, 2006 ▶).
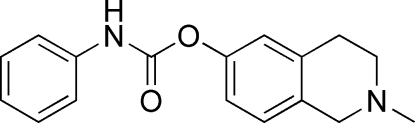


## Experimental

### 

#### Crystal data

C_17_H_18_N_2_O_2_
*M* *_r_* = 282.33
Monoclinic, 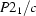

*a* = 6.0653 (6) Å
*b* = 15.5540 (17) Å
*c* = 15.1817 (16) Å
β = 93.488 (2)°
*V* = 1429.6 (3) Å^3^
*Z* = 4
Mo *K*α radiation
μ = 0.09 mm^−1^
*T* = 293 K
0.47 × 0.35 × 0.31 mm

#### Data collection

Bruker SMART CCD area-detector diffractometer
Absorption correction: multi-scan (*SADABS*; Sheldrick, 1996 ▶) *T*_min_ = 0.958, *T*_max_ = 0.963
7422 measured reflections
2662 independent reflections
2190 reflections with *I* > 2σ(*I*)
*R*_int_ = 0.087

#### Refinement

*R*[*F* ^2^ > 2σ(*F* ^2^)] = 0.046
*wR*(*F* ^2^) = 0.128
*S* = 1.02
2662 reflections
196 parameters
H atoms treated by a mixture of independent and constrained refinement
Δρ_max_ = 0.28 e Å^−3^
Δρ_min_ = −0.21 e Å^−3^

### 

Data collection: *SMART* (Bruker, 2000 ▶); cell refinement: *SAINT* (Bruker, 2000 ▶); data reduction: *SAINT*; program(s) used to solve structure: *SHELXS97* (Sheldrick, 2008 ▶); program(s) used to refine structure: *SHELXL97* (Sheldrick, 2008 ▶); molecular graphics: *SHELXTL* (Sheldrick, 2008 ▶); software used to prepare material for publication: *SHELXTL* and local programs.

## Supplementary Material

Crystal structure: contains datablocks global, I. DOI: 10.1107/S1600536809013415/rk2138sup1.cif

Structure factors: contains datablocks I. DOI: 10.1107/S1600536809013415/rk2138Isup2.hkl

Additional supplementary materials:  crystallographic information; 3D view; checkCIF report

## Figures and Tables

**Table 1:** Hydrogen-bond geometry (Å, °)

*D*—H⋯*A*	*D*—H	H⋯*A*	*D*⋯*A*	*D*—H⋯*A*
N1—H1⋯N2^i^	0.879 (16)	2.339 (16)	3.1886 (18)	162.5 (14)

Symmetry code: (i) 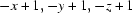.

**Fig. 1.**
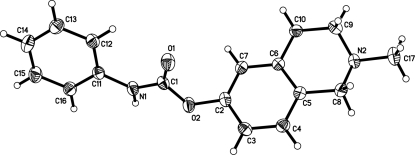
The molecular structure of title compound with the atom numbering scheme. The displacement ellipsoids are drawn at the 50% probability level. H atons are presented as a small spheres of arbitrary radius.

**Fig. 2.**
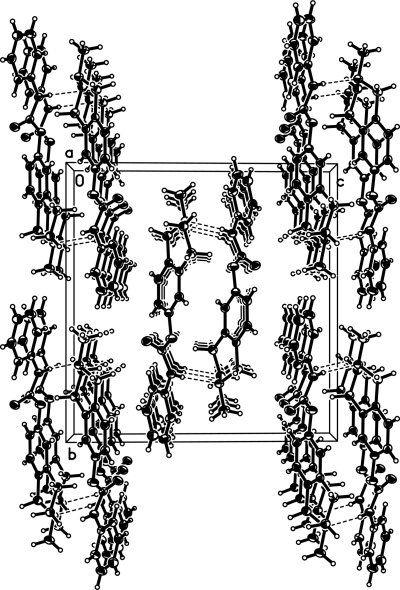
A view of the crystal packing, showing the hydrogen–bonding network.

## supplementary crystallographic information

### Comment

In the molecular structure of title compound (Fig.1), the piperidine ring adopts a half–chair form, with atoms N2 and C9 out of the plane defined by the remaining four atoms. The N1—C1 bond length [1.3485 (19) Å] is longer than that (1.32 Å) for a peptide linkage. The N1—C11 bond length [1.4128 (19) Å] is shorter than a normal C—N single bond and longer than a normal C═N bond, probably as a result of electron delocalization, suggesting that the N1—C11 bond participates in the conjugated system of the benzene ring (Li *et al.*, 2006). The two phenyl rings are planar and make a dihedral angle of 53.90°. The crystal structure is stabilized through intermolecular N1—H1···N2^i^ hydrogen bonds [symmetry code (i): 1-*x*, 1-*y,*, 1-*z*] and π–π stacking interactions (Fig.2).

### Experimental

The 2–methyl–1,2,3,4–tetrahydroisoquinolin–6–ol (6.13 mmol) was dissolved in anhydrous *THF* (100 ml), and a piece of Na metal (approximately 10 mg) was added. The mixture was stirred at room temperature for 15 min, then phenylisocyanate (18.48 mmol) was added. The reaction mixture was continuously stirred for 2 h at room temperature and monitored by *TLC*. The precipitate was filtered off and the filtrate was evaporated to give yellow oil. The 20 ml H_2_O was added and pH of the aqueous layer was adjusted to 3 by 1 *N* HCl, washed with *Et*_2_O, and then pH was adjusted to 10 by NaHCO_3 _aqueous solution (approximately 1%). The resulting precipitate was filtered and washed with water three times. A yellow solid (yield 1.50 g, 87%) was obtained, and single crystals suitable for crystallographic analysis were obtained by slow evaporation of an ethanol solution.

### Refinement

All C–bound H atoms were positioned geometrically and refined as riding (C—H = 0.93–0.97 Å), with *U*_iso_(H) = 1.2*U*_eq_(C) and the three H atoms of the methyl refined as riding (C—H = 0.98 Å), with *U*_iso_(H) = 1.5*U*_eq_(C). The H atom of the NH group was refined isotropically.

### Crystal data

**Table:**

C_17_H_18_N_2_O_2_	*F*(000) = 600
*M**_r_* = 282.33	*D*_x_ = 1.312 Mg m^−^^3^
Monoclinic, *P*2_1_/*c*	Mo *K*α radiation, λ = 0.71073 Å
Hall symbol: -P 2ybc	Cell parameters from 3056 reflections
*a* = 6.0653 (6) Å	θ = 5.2–55.0°
*b* = 15.5540 (17) Å	µ = 0.09 mm^−^^1^
*c* = 15.1817 (16) Å	*T* = 293 K
β = 93.488 (2)°	Block, yellow
*V* = 1429.6 (3) Å^3^	0.47 × 0.35 × 0.31 mm
*Z* = 4	

### Data collection

**Table:**

Bruker SMART CCD area-detector diffractometer	2662 independent reflections
Radiation source: Fine–focus sealed tube	2190 reflections with *I* > 2σ(*I*)
graphite	*R*_int_ = 0.087
φ and ω scans	θ_max_ = 25.5°, θ_min_ = 1.9°
Absorption correction: multi-scan (*SADABS*; Sheldrick, 1996)	*h* = −7→7
*T*_min_ = 0.958, *T*_max_ = 0.963	*k* = −18→13
7422 measured reflections	*l* = −18→18

### Refinement

**Table:**

Refinement on *F*^2^	Secondary atom site location: Difmap
Least-squares matrix: Full	Hydrogen site location: Geom
*R*[*F*^2^ > 2σ(*F*^2^)] = 0.046	H atoms treated by a mixture of independent and constrained refinement
*wR*(*F*^2^) = 0.128	*w* = 1/[σ^2^(*F*_o_^2^) + (0.067*P*)^2^] where *P* = (*F*_o_^2^ + 2*F*_c_^2^)/3
*S* = 1.01	(Δ/σ)_max_ < 0.001
2662 reflections	Δρ_max_ = 0.28 e Å^−^^3^
196 parameters	Δρ_min_ = −0.20 e Å^−^^3^
0 restraints	Extinction correction: *SHELXL97* (Sheldrick, 2008), Fc^*^=kFc[1+0.001xFc^2^λ^3^/sin(2θ)]^-1/4^
Primary atom site location: Direct	Extinction coefficient: 0.0090 (19)

### Special details

**Table:**

Geometry. All s.u.'s (except the s.u. in the dihedral angle between two l.s. planes) are estimated using the full covariance matrix. The cell s.u.'s are taken into account individually in the estimation of s.u.'s in distances, angles and torsion angles; correlations between s.u.'s in cell parameters are only used when they are defined by crystal symmetry. An approximate (isotropic) treatment of cell s.u.'s is used for estimating s.u.'s involving l.s. planes.
Refinement. Refinement of *F*^2^ against ALL reflections. The weighted *R*–factor *wR* and goodness of fit *S* are based on *F*^2^, conventional *R*–factors *R* are based on *F*, with *F* set to zero for negative *F*^2^. The threshold expression of *F*^2^ > σ(*F*^2^) is used only for calculating *R*–factors(gt) *etc*. and is not relevant to the choice of reflections for refinement. *R*–factors based on *F*^2^ are statistically about twice as large as those based on *F*, and *R*–factors based on ALL data will be even larger.

### Fractional atomic coordinates and isotropic or equivalent isotropic displacement parameters (Å^2^)

**Table:**

	*x*	*y*	*z*	*U*_iso_*/*U*_eq_	
N1	0.2614 (2)	0.25526 (8)	0.60923 (8)	0.0413 (3)	
N2	0.4681 (2)	0.78297 (8)	0.56012 (7)	0.0411 (3)	
O1	0.15429 (18)	0.36878 (7)	0.69300 (7)	0.0539 (3)	
O2	0.45190 (18)	0.37476 (6)	0.61019 (7)	0.0508 (3)	
C1	0.2728 (2)	0.33563 (10)	0.64250 (9)	0.0385 (4)	
C2	0.4760 (2)	0.46370 (9)	0.61918 (9)	0.0402 (4)	
C3	0.6718 (2)	0.49347 (10)	0.65786 (9)	0.0438 (4)	
H3	0.7760	0.4556	0.6831	0.053*	
C4	0.7104 (2)	0.58098 (10)	0.65837 (9)	0.0425 (4)	
H4	0.8422	0.6019	0.6846	0.051*	
C5	0.5568 (2)	0.63838 (9)	0.62065 (8)	0.0359 (3)	
C6	0.3585 (2)	0.60679 (9)	0.58205 (8)	0.0349 (3)	
C7	0.3195 (2)	0.51930 (10)	0.58201 (9)	0.0392 (4)	
H7	0.1872	0.4978	0.5568	0.047*	
C8	0.6021 (2)	0.73292 (10)	0.62511 (10)	0.0439 (4)	
H8A	0.5743	0.7534	0.6837	0.053*	
H8B	0.7570	0.7426	0.6159	0.053*	
C9	0.2354 (2)	0.76007 (10)	0.56625 (10)	0.0443 (4)	
H9A	0.1441	0.7981	0.5289	0.053*	
H9B	0.1946	0.7675	0.6266	0.053*	
C10	0.1941 (2)	0.66790 (10)	0.53803 (9)	0.0415 (4)	
H10A	0.0464	0.6513	0.5524	0.050*	
H10B	0.2018	0.6637	0.4745	0.050*	
C11	0.1091 (2)	0.19113 (9)	0.63109 (8)	0.0368 (3)	
C12	−0.0867 (2)	0.20917 (10)	0.66984 (9)	0.0430 (4)	
H12	−0.1227	0.2655	0.6834	0.052*	
C13	−0.2280 (3)	0.14259 (11)	0.68815 (10)	0.0490 (4)	
H13	−0.3596	0.1549	0.7139	0.059*	
C14	−0.1782 (3)	0.05886 (12)	0.66912 (11)	0.0555 (5)	
H14	−0.2737	0.0147	0.6826	0.067*	
C15	0.0147 (3)	0.04127 (11)	0.62988 (11)	0.0555 (4)	
H15	0.0488	−0.0151	0.6156	0.067*	
C16	0.1585 (3)	0.10679 (10)	0.61141 (10)	0.0456 (4)	
H16	0.2896	0.0941	0.5855	0.055*	
C17	0.4991 (3)	0.87447 (10)	0.57824 (12)	0.0585 (5)	
H17A	0.4182	0.9074	0.5337	0.088*	
H17B	0.6533	0.8884	0.5780	0.088*	
H17C	0.4464	0.8878	0.6350	0.088*	
H1	0.360 (3)	0.2430 (11)	0.5712 (10)	0.052 (5)*	

### Atomic displacement parameters (Å^2^)

**Table:**

	*U*^11^	*U*^22^	*U*^33^	*U*^12^	*U*^13^	*U*^23^
N1	0.0499 (8)	0.0326 (7)	0.0427 (7)	0.0008 (6)	0.0133 (6)	−0.0001 (5)
N2	0.0510 (8)	0.0318 (7)	0.0411 (7)	−0.0011 (5)	0.0071 (5)	−0.0010 (5)
O1	0.0678 (8)	0.0401 (7)	0.0561 (7)	−0.0014 (5)	0.0237 (6)	−0.0067 (5)
O2	0.0557 (7)	0.0331 (6)	0.0655 (7)	−0.0022 (5)	0.0203 (6)	−0.0032 (5)
C1	0.0460 (8)	0.0331 (8)	0.0365 (7)	0.0036 (7)	0.0036 (6)	0.0051 (6)
C2	0.0511 (9)	0.0318 (8)	0.0388 (7)	−0.0005 (7)	0.0119 (6)	−0.0001 (6)
C3	0.0482 (9)	0.0428 (9)	0.0399 (8)	0.0067 (7)	−0.0005 (6)	0.0041 (6)
C4	0.0415 (8)	0.0469 (10)	0.0383 (8)	−0.0022 (7)	−0.0036 (6)	−0.0009 (7)
C5	0.0393 (8)	0.0378 (8)	0.0309 (7)	−0.0018 (6)	0.0043 (6)	−0.0017 (6)
C6	0.0359 (8)	0.0380 (8)	0.0309 (7)	−0.0011 (6)	0.0044 (6)	−0.0009 (6)
C7	0.0397 (8)	0.0398 (9)	0.0381 (7)	−0.0057 (6)	0.0028 (6)	−0.0033 (6)
C8	0.0430 (8)	0.0411 (9)	0.0471 (8)	−0.0048 (7)	−0.0004 (6)	−0.0048 (7)
C9	0.0473 (9)	0.0429 (9)	0.0425 (8)	0.0075 (7)	0.0023 (6)	0.0006 (7)
C10	0.0377 (8)	0.0441 (9)	0.0424 (8)	0.0007 (7)	0.0010 (6)	0.0027 (7)
C11	0.0434 (8)	0.0365 (8)	0.0301 (7)	0.0010 (6)	0.0007 (6)	0.0034 (6)
C12	0.0500 (9)	0.0398 (9)	0.0395 (8)	0.0041 (7)	0.0058 (6)	0.0024 (6)
C13	0.0480 (9)	0.0564 (11)	0.0430 (8)	−0.0065 (8)	0.0068 (7)	0.0005 (7)
C14	0.0618 (11)	0.0503 (11)	0.0545 (10)	−0.0188 (8)	0.0045 (8)	−0.0017 (8)
C15	0.0658 (11)	0.0366 (9)	0.0640 (10)	−0.0070 (8)	0.0025 (9)	−0.0095 (8)
C16	0.0483 (9)	0.0401 (9)	0.0487 (8)	0.0003 (7)	0.0052 (7)	−0.0057 (7)
C17	0.0793 (13)	0.0361 (9)	0.0611 (10)	−0.0047 (8)	0.0132 (9)	−0.0054 (8)

### Geometric parameters (Å, °)

**Table:**

N1—C1	1.3485 (19)	C8—H8B	0.9700
N1—C11	1.4128 (19)	C9—C10	1.513 (2)
N1—H1	0.879 (16)	C9—H9A	0.9700
N2—C17	1.4596 (19)	C9—H9B	0.9700
N2—C8	1.4636 (18)	C10—H10A	0.9700
N2—C9	1.464 (2)	C10—H10B	0.9700
O1—C1	1.1989 (17)	C11—C16	1.382 (2)
O2—C1	1.3622 (17)	C11—C12	1.385 (2)
O2—C2	1.3970 (18)	C12—C13	1.383 (2)
C2—C3	1.373 (2)	C12—H12	0.9300
C2—C7	1.379 (2)	C13—C14	1.372 (2)
C3—C4	1.381 (2)	C13—H13	0.9300
C3—H3	0.9300	C14—C15	1.372 (2)
C4—C5	1.389 (2)	C14—H14	0.9300
C4—H4	0.9300	C15—C16	1.381 (2)
C5—C6	1.395 (2)	C15—H15	0.9300
C5—C8	1.497 (2)	C16—H16	0.9300
C6—C7	1.381 (2)	C17—H17A	0.9600
C6—C10	1.505 (2)	C17—H17B	0.9600
C7—H7	0.9300	C17—H17C	0.9600
C8—H8A	0.9700		
			
C1—N1—C11	125.86 (13)	N2—C9—H9A	109.5
C1—N1—H1	115.2 (11)	C10—C9—H9A	109.5
C11—N1—H1	118.9 (11)	N2—C9—H9B	109.5
C17—N2—C8	109.37 (12)	C10—C9—H9B	109.5
C17—N2—C9	109.81 (12)	H9A—C9—H9B	108.1
C8—N2—C9	109.00 (12)	C6—C10—C9	112.26 (12)
C1—O2—C2	119.22 (11)	C6—C10—H10A	109.2
O1—C1—N1	128.26 (14)	C9—C10—H10A	109.2
O1—C1—O2	124.08 (14)	C6—C10—H10B	109.2
N1—C1—O2	107.64 (12)	C9—C10—H10B	109.2
C3—C2—C7	121.30 (13)	H10A—C10—H10B	107.9
C3—C2—O2	117.29 (13)	C16—C11—C12	119.11 (14)
C7—C2—O2	121.04 (13)	C16—C11—N1	117.77 (14)
C2—C3—C4	118.48 (13)	C12—C11—N1	123.12 (14)
C2—C3—H3	120.8	C13—C12—C11	119.42 (15)
C4—C3—H3	120.8	C13—C12—H12	120.3
C3—C4—C5	121.47 (14)	C11—C12—H12	120.3
C3—C4—H4	119.3	C14—C13—C12	121.43 (16)
C5—C4—H4	119.3	C14—C13—H13	119.3
C4—C5—C6	119.11 (14)	C12—C13—H13	119.3
C4—C5—C8	119.75 (12)	C13—C14—C15	119.02 (16)
C6—C5—C8	121.09 (13)	C13—C14—H14	120.5
C7—C6—C5	119.39 (13)	C15—C14—H14	120.5
C7—C6—C10	120.88 (13)	C14—C15—C16	120.44 (16)
C5—C6—C10	119.70 (13)	C14—C15—H15	119.8
C2—C7—C6	120.26 (13)	C16—C15—H15	119.8
C2—C7—H7	119.9	C15—C16—C11	120.57 (15)
C6—C7—H7	119.9	C15—C16—H16	119.7
N2—C8—C5	113.52 (11)	C11—C16—H16	119.7
N2—C8—H8A	108.9	N2—C17—H17A	109.5
C5—C8—H8A	108.9	N2—C17—H17B	109.5
N2—C8—H8B	108.9	H17A—C17—H17B	109.5
C5—C8—H8B	108.9	N2—C17—H17C	109.5
H8A—C8—H8B	107.7	H17A—C17—H17C	109.5
N2—C9—C10	110.88 (12)	H17B—C17—H17C	109.5
			
C11—N1—C1—O1	−3.2 (2)	C17—N2—C8—C5	171.44 (13)
C11—N1—C1—O2	175.23 (12)	C9—N2—C8—C5	51.38 (16)
C2—O2—C1—O1	−14.8 (2)	C4—C5—C8—N2	161.06 (13)
C2—O2—C1—N1	166.71 (12)	C6—C5—C8—N2	−21.39 (19)
C1—O2—C2—C3	126.45 (14)	C17—N2—C9—C10	173.79 (12)
C1—O2—C2—C7	−60.49 (18)	C8—N2—C9—C10	−66.42 (15)
C7—C2—C3—C4	−0.4 (2)	C7—C6—C10—C9	164.33 (13)
O2—C2—C3—C4	172.59 (12)	C5—C6—C10—C9	−18.03 (18)
C2—C3—C4—C5	−0.2 (2)	N2—C9—C10—C6	48.87 (16)
C3—C4—C5—C6	0.5 (2)	C1—N1—C11—C16	−161.74 (14)
C3—C4—C5—C8	178.10 (13)	C1—N1—C11—C12	19.2 (2)
C4—C5—C6—C7	−0.1 (2)	C16—C11—C12—C13	0.1 (2)
C8—C5—C6—C7	−177.68 (13)	N1—C11—C12—C13	179.23 (13)
C4—C5—C6—C10	−177.79 (12)	C11—C12—C13—C14	0.3 (2)
C8—C5—C6—C10	4.6 (2)	C12—C13—C14—C15	−1.0 (2)
C3—C2—C7—C6	0.8 (2)	C13—C14—C15—C16	1.2 (2)
O2—C2—C7—C6	−171.95 (12)	C14—C15—C16—C11	−0.8 (2)
C5—C6—C7—C2	−0.5 (2)	C12—C11—C16—C15	0.1 (2)
C10—C6—C7—C2	177.12 (12)	N1—C11—C16—C15	−179.00 (13)

### Hydrogen-bond geometry (Å, °)

**Table:**

*D*—H···*A*	*D*—H	H···*A*	*D*···*A*	*D*—H···*A*
N1—H1···N2^i^	0.879 (16)	2.339 (16)	3.1886 (18)	162.5 (14)

Symmetry codes: (i) −*x*+1, −*y*+1, −*z*+1.
